# The Other Half of the Risk: Including Men in Valproate Safety Practice (An Audit of Compliance With Updated National Valproate Prescribing Guidelines)

**DOI:** 10.1192/bjo.2026.11825

**Published:** 2026-06-30

**Authors:** Arezu Pourahmad Nodehi, Cathy Carter, Craig Horner

**Affiliations:** 1 Kent and Medway Mental Health NHS Trust, Maidstone, United Kingdom; 2 Oxleas NHS Foundation Trust, Bromley, United Kingdom; 3 Barnard Medical Group, Sidcup, United Kingdom

## Abstract

**Aims:**

Sodium valproate safety protocols historically focused exclusively on women of childbearing potential. However, 2024 Medicines and Healthcare products Regulatory Agency (MHRA) alerts represent a significant paradigm shift, extending safety requirements to include male patients due to emerging evidence of potential risks regarding male infertility and offspring congenital and neuro developmental disorders.

Recognising an ethical duty to ensure transparency and patient autonomy, the East Bromley Intensive Case Management in Psychosis (ICMP) team conducted this audit to:

**Establish a clinical registry** to monitor all valproate prescribing within the service.

**Assess baseline compliance** with national standards (risk communication, contraception advice, informed consent).

**Deploy targeted interventions** to strengthen shared decision-making and ensure regulatory adherence across all genders.

**Methods:**

**Cycle 1:** A retrospective review of Electronic Patient Records for 330 patients was performed. A registry was established for all patients prescribed sodium valproate. Baseline data captured adherence to updated guidance, including documentation of risk discussions, contraception advice, informed consent, and Annual Risk Acknowledgement Form (ARAF) completion.

**Interventions:** Targeted interventions included clinician education, structured outpatient reviews (26 conducted), distribution of MHRA materials, and the creation of a live ARAF tracking system.

**Cycle 2:** A focused re-audit in July 2025 evaluated the impact of these interventions.

**Results:**

A registry of 46 patients was established (30 Males(M), 5 Females(F)<55, 11 F>55).

**Baseline Compliance (Cycle 1):** Data revealed a marked gender disparity in regulatory adherence, with male patients significantly falling behind:

- Risk Discussions: 37% (F:80%, M:17%)
- Contraception Advice: 15% (F:80%, M:3%)
- Informed Consent: 61% (F:80%, M:20%)
- MHRA Material Provision: 11% (F:60%, M:6%)

Dual-signed ARAF in eligible females: 0%

**Post-Intervention (Cycle 2):** Targeted interventions successfully bridged this gap, achieving marked improvements in the male cohort:

- Risk Discussions: 17%→87%
- Contraception Advice: 3%→83%
- Informed Consent: 20%→87%
- MHRA Materials: 6%→83%

The pathway for second-consultant sign-off was clarified, with 100% of eligible females reviewed.

Furthermore, 26 structured reviews directly influenced prescribing: 13% of patients transitioned to alternative treatments, and 9% deliberating alternatives, reflecting a significant shift toward active shared decision-making.

**Conclusion:**

This project exposed and successfully closed a critical safety gap in male valproate prescribing by deploying targeted risk discussions and active clinical re-evaluation. The 13% treatment switch rate underscores the impact of shared decision-making, proving that informed consent is an evolving dialogue rather than a static achievement. Ultimately, this work reaffirms the clinicians’ ethical duty to challenge long-standing treatment plans in light of new evidence, ensuring psychiatric practice remains both evidence-based and ethically robust.

